# Serological and molecular screening of arenaviruses in suspected tick-borne encephalitis cases in Finland

**DOI:** 10.1017/S0950268824000128

**Published:** 2024-01-22

**Authors:** Hussein Abas Thamer Alburkat, Emilia Pulkkinen, Jenni Virtanen, Olli Vapalahti, Tarja Sironen, A. J. Jääskeläinen

**Affiliations:** 1 Department of Virology, Faculty of Medicine, University of Helsinki, Helsinki, Finland; 2 Department of Veterinary Biosciences, University of Helsinki, Helsinki, Finland; 3 HUS Diagnostic Center, Helsinki University Hospital, and University of Helsinki, Helsinki, Finland; 4 ISLAB Laboratory Centre, Kuopio, Finland

**Keywords:** arenaviruses, Serology, surveillance, LCMV-like virus, zoonoses

## Abstract

Lymphocytic choriomeningitis virus (LCMV) is one of the arenaviruses infecting humans. LCMV infections have been reported worldwide in humans with varying levels of severity. To detect arenavirus RNA and LCMV-reactive antibodies in different geographical regions of Finland, we screened human serum and cerebrospinal fluid (CSF) samples, taken from suspected tick-borne encephalitis (TBE) cases, using reverse transcriptase polymerase chain reaction (RT-PCR) and immunofluorescence assay (IFA). No arenavirus nucleic acids were detected, and the overall LCMV seroprevalence was 4.5%. No seroconversions were detected in paired serum samples. The highest seroprevalence (5.2%) was detected among individuals of age group III (40–59 years), followed by age group I (under-20-year-olds, 4.9%), while the lowest seroprevalence (3.8%) was found in age group IV (60 years or older). A lower LCMV seroprevalence in older age groups may suggest waning of immunity over time. The observation of a higher seroprevalence in the younger age group and the decreasing population size of the main reservoir host, the house mouse, may suggest exposure to another LCMV-like virus in Finland.

## Introduction

One of the arenaviruses infecting humans is the lymphocytic choriomeningitis virus (LCMV), which is a segmented negative-sense single-stranded RNA virus within the *Mammarenavirus* genus, family Arenaviridae. LCMV genome is composed of two RNA segments: a large segment (L-segment) and a small segment (S-segment). The L-segment is a highly conserved segment that encodes for the L-protein and Z-protein, while the S segment encodes for the nucleoprotein (NP) and glycoprotein complex (GPC) [1]. Humans are thought to acquire LCMV infection through direct contact with rodents, rodent bites, or mucosal exposure to aerosols contaminated with rodent excreta. In humans, the clinical picture of LCMV infection ranges from asymptomatic to severe manifestations, such as aseptic meningitis, severe systemic infections in immunocompromised persons, and congenital abnormalities [2]. Typically, a biphasic disease can occur with unspecific first-phase symptoms including flu-like illness with fever, followed by a second phase with more neurological symptoms mimicking, for example, tick-borne encephalitis virus (TBEV) infection [3]. Immunosuppressed individuals and transplant recipients are the most vulnerable to LCMV infection, in addition to pregnant individuals, who are prone to fatal infections [4–6]. For LCMV, the common house mouse (*Mus musculus*) is the reservoir host. However, serological data indicate that the LCMV or LCMV-like viruses can also infect pet mice, hamsters (*Mesocricetus auratus*), forest voles (*Microtus agrestis*), and Guinea pigs (*Cavia porcellus*) [3, 7–9]. Furthermore, LCMV-like virus RNA was detected in wood mice (*Apodemus sylvaticus*) in Spain, suggesting that a new lineage of LCMV or a LCMV-like virus circulates among wood mice [10]. In Australia, during an investigation of a small cluster of transplant-related fatal cases, an LCMV variant, Dandenong virus, was discovered [11].

Studies conducted in the USA in 1953 and 1992 reported the presence of antibodies against LCMV in 8% of neuroinvasive patients and 5.1% of the healthy, respectively [12, 13]. In Argentina, LCMV is endemic in some cities, and there are a few studies reporting LCMV seroprevalence rates of 2.3% and 1–3.6% in the 1990s and between 1998 and 2003, respectively [14, 15]. In Europe, Tagliapietra et al. [16] showed that the seroprevalence increased from 2.5% to 7% among Italian forest workers between 2002 and 2015 in the province of Trento. Another study from France showed that only 1.7% of the population was positive for anti-LCMV antibodies [17]. However, on Vir Island in Croatia, a high seroprevalence for LCMV (36%) has been reported [18]. In this Croatian study, the IFA cut-off was relatively lower, which might have affected the results. However, in some countries, LCMV may be more prevalent. In Finland, a seroprevalence of 5% has been previously reported for LCMV [19], and this is in line with most European countries. In addition to human data, there is evidence of arenavirus-reactive antibodies in Finnish field voles (2.4%) [8], but no LCMV or other arenavirus nucleic acids have been detected so far, in humans or rodents, in Finland. Currently, there are no studies on screening of pet rodents or other wild rodents, while screening studies on field voles in Finland are available. Interestingly, using next-generation sequencing (NGS), Sanchez Romano et al. (2021) [20] detected reads similar to Arenaviridae from tundra reindeer (*Rangifer tarandus tarandus*) in northern Finland. In this study, we aimed to detect arenavirus nucleic acids in available human samples [serum and cerebrospinal fluid (CSF)] and to study LCMV or LCMV-like virus seroprevalence in different geographical regions of Finland. Previously, 400 patients suspected of having a central nervous system infection were examined for LCMV infections [19], but in this study, we aimed to study samples with a wider distribution using samples taken from suspected tick-borne encephalitis (TBE) cases, as the disease profile mimics LCMV infection, and to detect the LCMV from these human samples. Furthermore, we aimed to highlight some factors that are associated with varied degrees of arenavirus seroprevalence between regions.

## Materials and methods

### Samples

**Figure 1.**
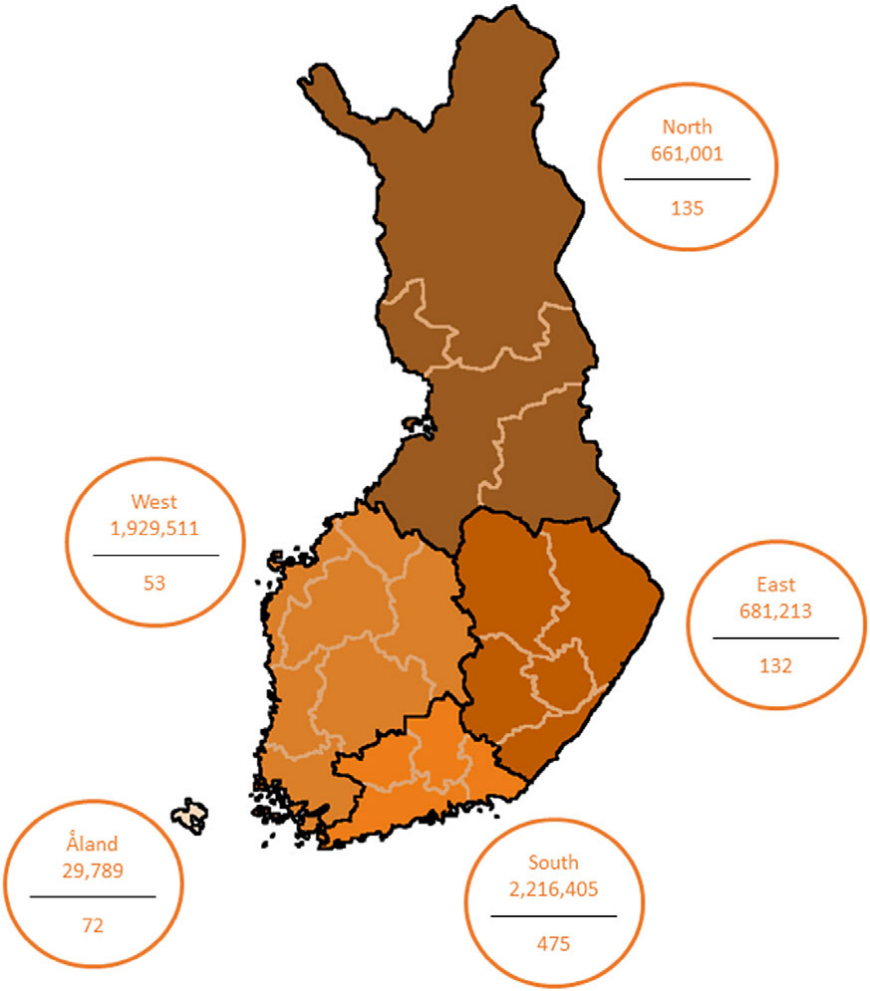
Map of Finland illustrating the regions, total population (above line), and number of individuals included in the study (below line).

As symptoms of LCMV infection can mimic those of TBEV infection, samples sent to the Helsinki University Hospital (Diagnostic Center, HUS, Helsinki, Finland) for anti-TBEV antibody detection could be considered potentially enriched for LCMV. All serum and CSF samples from the high season of TBE – June to November 2018 (total of 6 months) – which were sent for screening of anti-TBEV antibodies to the Diagnostic Center (HUS, Helsinki, Finland), were included in this study, including in a total of 976 serum samples from 867 individuals aged 1–89 (mean 46) years (Table 1). Of these 867 individuals, 222 had corresponding CSF samples available (a total of 227 CSFs), and all of them were included in this study. All samples were recoded and treated anonymously. Information on gender was not available. This study was approved by the Research Administration of the Helsinki University Hospital (HUS/32/2018, HUS/157/2020, HUS/151/2022). Five different geographical regions following the borderlines of Finland’s hospital districts in 2018 were included in this study: north, east, west, south, and Åland (Figure 1).

**Table 1.** Age distribution and population parameters within the five regions

	Whole population in Finland, 2018^a^			This study, 2018			
Region	Population (n)	Population density (/km^2^)	Mean age (years)	Individuals (n)	Age (mean/median)	Age (SD)	Age (range)
North	661,001	6	44	135	41/42	26	1–87
East	681,213	14	47	132	53/59	23	3–87
West	1,929,511	29	40	53	49/55	22	4–88
South	2,216,405	74	44	475	45/46	22	1–89
Åland	29,789	19	43	72	46/46	23	3–88
Total	5,517,919	18.2	42.9	867	46/49	23	1–89

a Reference to the database concerning the population data [24].

### Immunofluorescence assay

All 867 individuals (976 serum samples) were screened for anti-LCMV IgG antibodies using immunofluorescence assay (IFA), as described previously [19, 21, 22]. The IFA method is based on the LCMV Armstrong strain-infected Vero E6 cells, and a serum dilution of 1:20 was used. There are both LCMV-infected and non-infected Vero-E6 cells in all wells of the IFA slide. The non-infected cells are in-well internal controls for the detection of unspecific reactivities. Our method is not only specific to LCMV but is also based on whole LCMV, enabling the detection of LCMV-like viruses. After the detection of anti-LCMV IgG antibodies, all the positive samples for anti-LCMV IgG were tested for anti-LCMV IgM antibodies, as described previously [19]. All the samples were stored at −80 °C until use. Antibodies were not screened from the CSF samples.

## Detection of viral RNA and sequencing

Nucleic acids were extracted from 976 serum samples using the MagNA Pure LC system, and the Total Nucleic Acid Kit (Roche) and NucliSENS easyMAG (bioMérieux) were used for the 227 CSF samples following the manufacturers’ instructions. In addition, the Qiagen Viral Mini Kit was used for the re-extraction of 49 serum and 19 CSF samples. The extracted nucleic acids were used as templates in RT-PCR experiments targeting the conserved L gene of Old-World arenaviruses using primers designed by Vieth et al. [23]. The RT-PCR-mix included the Invitrogen SuperScript®III One-Step RT-PCR System with Platinum®Taq DNA Polymerase (Invitrogen, Carlsbad, CA) and primers of 320 nm LVL_3359D, 320 nm LVL_3359G, 480 nm LVL_3754A, and 480 nm LVL_3754D with 4 μL of template in a total volume of 20 μL. RT-PCR included a 30-min reverse transcription phase at 50 °C, followed by 2 min at 94 °C, and 45 cycles of 20 sec at 94°C, 30 at sec 55°C and 1 min at 72 °C, with a final elongation step of 5 min at 72 °C. PCR products were visualized using agarose gel electrophoresis (2.2%), and the PCR products [size over 350 base pairs (bp)] from patient samples, in addition to the positive control (LCMV Armstrong strain), were purified using the QuickStep™ 2 PCR Purification Kit (EdgeBio), followed by sequencing at the Diagnostic Center (HUS, Helsinki, Finland) using the Sanger sequencing platform.

### Statistical analysis

Statistical analysis was carried out using IBM SPSS Statistics version 28 [24]. Differences between independent groups of continuous variables were tested using the independent-samples Mann–Whitney U test (two groups) or independent-samples Kruskal–Wallis test (multiple groups). Normality of the data was tested using the Shapiro–Wilk test, histograms, and Q-Q plots. Categorical data were tested using the Fisher–Freeman–Halton exact test. P-values below 0.05 were considered statistically significant, and confidence intervals were calculated using the Wilson method. Population data were acquired from Statistics Finland’s free-of-charge databases [25].

## Results

No arenavirus nucleic acids were detected from 976 serum and 227 CSF samples studied. Although some PCR products were detected, when sequenced, no LCMV sequences were detected among the tested samples. In addition, no seroconversions were detected from paired serum samples taken from a total of 60 individuals; all were either negative or positive for anti-LCMV IgG antibodies in both paired samples. All anti-LCMV IgG-positive samples were negative for anti-LCMV IgM.

The overall LCMV seroprevalence was 4.5% (95% CI 3.3–6.1%) (June–November 2018). The highest LCMV seroprevalence was detected among individuals of age group I (40–59 years) (5.2%) and the lowest in age group IV (60 years or older) (3.8%) (Table 2). LCMV seroprevalences detected in individuals of age groups I and II (under the age of 20 years and 20–39 years) were 4.9% and 4.4%, respectively (Table 2). The LCMV seroprevalence was slightly higher in the north region (6.7%), followed by Åland (5.6%) and south regions (5.1%), respectively (Table 2). Statistical analysis showed slight differences in the age distribution between regions (Supplementary Figure 1, Supplementary Table 1 available on the Cambridge Core website), with the median age group being 40–60 years (p = 0.001 between south and east and north and east). Overall, the difference between age and IgG status was not statistically significant (p-value = 0.465), but there were some variations in the seroprevalence between different regions. Anti-LCMV antibodies were detected in all the regions, with the highest percentage in the north region and the lowest in the east region.

**Table 2.** Overall seroprevalence of arenavirus-reactive antibodies detected in human samples according to their age groups and geographical regions. The IFA seropositive shown in percentages

	Count (positive/all samples)	Seroprevalence (%)	95% CI
Group I (<20)	8/163	4.9	2.5–9.4
Group II (20–39)	8/183	4.4	2.2–8.4
Group III (40–59)	12/233	5.2	3.0–8.8
Group IV (>60)	11/289	3.8	2.1–6.7
East	1/132	0.8	0.1–4.2
North	9/135	6.7	3.6–12.2
South	24/475	5.1	3.4–7.4
West	1/53	1.9	0.3–10.0
Åland	4/72	5.6	2.2–13.4

## Discussion

**Figure 2.**
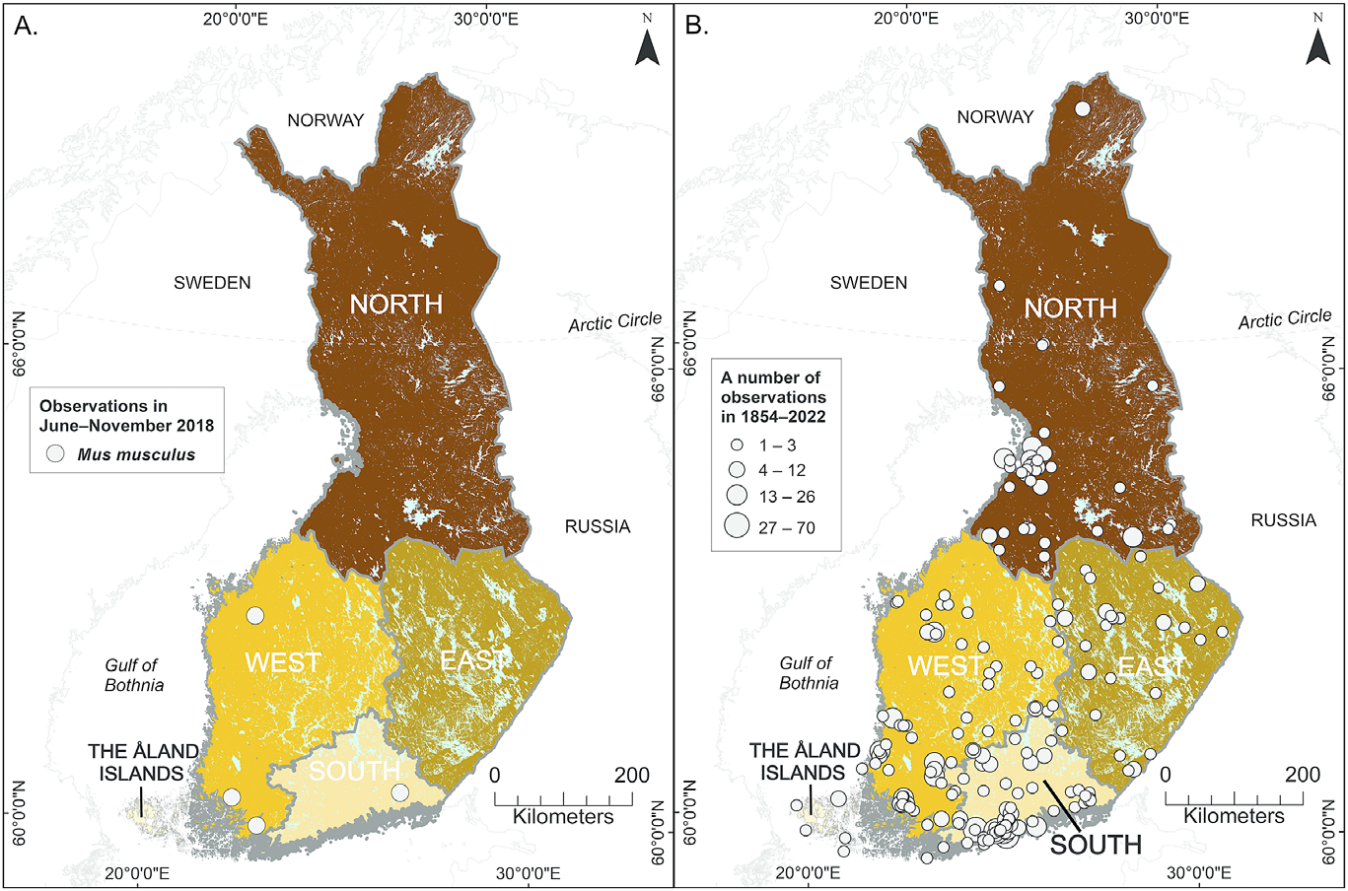
*Mus musculus* observations in Finland. (a) Observation of *M. musculus* in Finland from 1854 to 2022. (b) Observation of *M. musculus* in Finland during the study period June–November 2018. Finnish Biodiversity Info Facility [29].

In this study, we performed a survey for LCMV prevalence in humans using molecular and serological assays. A total of 867 individuals were screened for LCMV or LCMV-like virus nucleic acids and LCMV-reactive antibodies. In contrast to a previous study [19], we screened samples originating from cases suspected of having TBE with altogether more geographical variation within Finland. However, data from these individuals do not represent the general population, and only suggestions can be drawn. The overall seroprevalence detected in our study (4.5%) is in line with that in the previous study (5%) [19]. In terms of geographical distribution, LCMV seroprevalence varied between the regions, with northern Finland having the highest seroprevalence (6.7%) and eastern Finland the lowest (0.8%) (Supplementary Figure 1). The highest LCMV seroprevalence in northern Finland is likely associated with unidentified factors; for instance, exposure to different host species might be high. For example, Sanchez Romano et al. (2021) [20] reported that the most abundant reads were found for Arenaviridae samples using NGS that were taken from tundra reindeer of northern Finland. This raises the following question: Is there an unknown arenavirus in reindeer, or Is this something detected from the reindeer genome? There were no LCMV–host (*M. musculus*) observations in the north region during the study period (Figure 2). Interestingly, the highest LCMV seroprevalence was detected in the younger age group. This highlights the likelihood of divergent species acting as hosts for LCMV or LCMV-like viruses. In one study conducted in Finland, the presence of anti-LCMV antibodies has also been reported in Finnish field voles (*M. agrestis*) [8]. Unfortunately, no other studies are available from Finland.

The detection of LCMV-like arenavirus in small mammals other than *M. musculus* has been reported worldwide. In Spain, LCMV RNA was detected in wood mice (*A. sylvaticus*), and the results revealed a new lineage of LCMV, indicating the presence of a newly identified LCMV-like virus [10]. Probably, there might be many more arenaviruses to be found in rodents. Indeed, antibodies against LCMV or LCMV-like arenavirus could be detected in different rodent species, but without viral sequence data, it is difficult to speculate whether it is the LCMV or a LCMV-like virus. As the immunofluorescence method used in this study is based on the whole virus antigen, cross-reactions to closely related viruses might have occurred. Moreover, cross-reactions could happen between Old-World and New-World arenaviruses; for instance, mice immunized with Old-World arenavirus (Lassa and Mopeia viruses) showed antibodies cross-reactive to New-World arenaviruses (South American arenaviruses) [26].

In general, the estimated time of the viremia in LCMV patients is approximately 2 weeks after the first symptoms (first phase). Antibodies that can neutralize the virus typically develop 2–6 weeks after the onset of symptoms and can persist for a period of 6 months to 5 years [27]. However, Bond et al. (2013) [28] have suggested that anti-Lassa virus IgG antibodies can persist even up to 40 years. Virus titres in the CSF are lower and are present for a shorter time, which makes the detection of the virus difficult. Nevertheless, molecular methods carried out on CSF samples are still considered the most reliable method for LCMV detection, and LCMV RNA from CSF was characterized in previous studies [4, 29]. Serology can only point out potential recent infection [3]; the sample is usually positive for both anti-LCMV IgG and IgM antibodies. We also looked for acute infections by searching for seroconversions by paired samples but found none.

The house mouse (*M. musculus*) is considered to be the host for LCMV, but there is evidence that other rodents can also have anti-LCMV (or anti-LCMV-like virus) antibodies [8], [17]. In Finland, the range of *M. musculus* seems to be diminishing, and nowadays, the species is reported only occasionally by the Finnish Biodiversity Info Facility [30], which is an open-access data source that provides data on observations of certain species in Finland. However, these data cannot be considered true markers of population sizes in Finland. Curiously, in this study, LCMV seroprevalence is a little higher in younger human age groups. Based on our study and the knowledge of decreased *M. musculus* observations in Finland, it seems that those in age group III (40–59 years) and young people were in contact with the virus at the same level or even more than older people, raising questions on the source of infection (rodent) or contact with the LCMV or LCMV-like viruses. But as our data are not based on the general population, more data are needed. However, the precise arenavirus type or types present in Finland remains unconfirmed [18]. In future, studies on different Finnish rodents, perhaps including pet rodents like hamsters, should be carried out to discover whether there are some LCMV-related viruses.

## Conclusion

In this study, a survey was performed to detect arenavirus nucleic acids in humans and to study LCMV seroprevalence in different geographical regions of Finland. The results demonstrate that anti-LCMV antibodies were fairly common, and in this study, there were more seropositive cases in younger age groups than in old age groups. The coincidence of the diminishing range of *M. musculus* and LCMV seroprevalence in young age groups suggests the exposure of an LCMV-like virus in Finland.

## Supporting information

Alburkat et al. supplementary material 1 Alburkat et al. supplementary material

Alburkat et al. supplementary material 2 Alburkat et al. supplementary material

## Data Availability

The data that support the findings of this study are available upon request.

## Supplementary material

The supplementary material for this article can be found at http://doi.org/10.1017/S0950268824000128.

## References

[1] Lee KJ, et al. (2000) NP and L proteins of lymphocytic choriomeningitis virus (LCMV) are sufficient for efficient transcription and replication of LCMV genomic RNA analogs. Journal of Virology 74, 3470–3477. 10729120 10.1128/jvi.74.8.3470-3477.2000 PMC111854

[2] Bonthius DJ (2012) Lymphocytic choriomeningitis virus: An underrecognized cause of neurologic disease in the fetus, child, and adult. Seminars in Pediatric Neurology 19, 89–95. 22889536 10.1016/j.spen.2012.02.002 PMC4256959

[3] Stone GS, et al. (2019) Case 40-2019: A 26-year-old returning traveler with headache. New England Journal of Medicine 381, 2553–2560. 31881142 10.1056/NEJMcpc1904042

[4] Fischer SA, et al. (2006) Transmission of lymphocytic choriomeningitis virus by organ transplantation. New England Journal of Medicine 354, 2235–2249. 16723615 10.1056/NEJMoa053240

[5] Lapošová K, et al. (2016) Development and application of ELISA for the detection of IgG antibodies to lymphocytic choriomeningitis virus. Acta Virologica 60, 143–150. 27265463 10.4149/av_2016_02_143

[6] Delaine M, et al. (2017) Microcephaly caused by lymphocytic choriomeningitis virus. Emerging Infectious Diseases 23, 1548–1550. 28820372 10.3201/eid2309.170775 PMC5572864

[7] Martínez Peralta LA, et al. (1990) Infection of Guinea pigs with two strains of lymphocytic choriomeningitis virus. Medicina Buenos Aires 50, 225–229. 2130208

[8] Forbes KM, et al. (2014) Serological survey of rodent-borne viruses in Finnish field voles. Vector Borne Zoonotic Diseases Journal 14, 278–283. 10.1089/vbz.2013.1526 PMC3993079 24689532

[9] Biggar RJ, et al. (1975) Lymphocytic choriomeningitis outbreak associated with pet hamsters: Fifty-seven cases from New York state. Journal of the American Medical Association 232, 494–500. 1173141

[10] Ledesma J, et al. (2009) Independent lineage of lymphocytic choriomeningitis virus in wood mice (Apodemus sylvaticus), Spain. Emerging Infectious Diseases 15, 1677–1680. 19861074 10.3201/eid1510.090563 PMC2866409

[11] Palacios G, et al. (2008) A new arenavirus in a cluster of fatal transplant-associated diseases. New England Journal of Medicine 358, 991–998. 18256387 10.1056/NEJMoa073785

[12] Adair CV, Gauld RL and Smadel JE (1953) Aseptic meningitis, a disease of diverse etiology: Clinical and etiologic studies on 854 cases. Annals of Internal Medicine 39, 675–704. 13092736 10.7326/0003-4819-39-4-675

[13] Stephensen CB, et al. (1992) Prevalence of serum antibodies against lymphocytic choriomeningitis virus in selected populations from two U.S. cities. Journal of Medical Virology 38, 27–31. 1402829 10.1002/jmv.1890380107

[14] Ambrosio A, et al. (2014) Ecological and epidemiological features of lymphocytic choriomeningitis virus activity in Argentina. Current Opinion in Virology 12, 53–63.

[15] Riera L, et al. (2005) Serological study of the lymphochoriomeningitis virus (LCMV) in an inner city of Argentina. Journal of Medical Virology 76, 285–289. 15834871 10.1002/jmv.20357

[16] Tagliapietra V, et al. (2018) Emerging rodent-borne viral zoonoses in Trento, Italy. EcoHealth 15, 695–704. 29796719 10.1007/s10393-018-1335-4

[17] Lledó L, et al. (2003) Lymphocytic choriomeningitis virus infection in a province of Spain: Analysis of sera from the general population and wild rodents. Journal of Medical Virology 70, 273–275. 12696116 10.1002/jmv.10389

[18] Dobec M, et al. (2006) High prevalence of antibodies to lymphocytic choriomeningitis virus in a murine typhus endemic region in Croatia. Journal of Medical Virology 78, 1643–1647. 17063527 10.1002/jmv.20749

[19] Fevola C, et al. (2018) Seroprevalence of lymphocytic choriomeningitis virus and Ljungan virus in Finnish patients with suspected neurological infections. Journal of Medical Virology 90, 429–435. 28976562 10.1002/jmv.24966

[20] Sánchez Romano J, et al. (2021) Screening of Eurasian tundra reindeer for viral sequences by next-generation sequencing. International Journal of Environmental Research and Public Health 18(12) 6561. 10.3390/ijerph18126561. 34207171 PMC8296488

[21] Kallio-Kokko H, et al. (2006) Hantavirus and arenavirus antibody prevalence in rodents and humans in Trentino, Northern Italy. Epidemiology and Infection 134, 830–836. 16371172 10.1017/S0950268805005431 PMC2870443

[22] Jääskeläinen AJ, et al. (2008) Evidence of Ljungan virus specific antibodies in humans and rodents, Finland. Journal of Medical Virology 85, 2001–2008. 10.1002/jmv.23681 23852812

[23] Vieth S, et al. (2005) RT-PCR assay for detection of Lassa virus and related old world arenaviruses targeting the L gene. Royal Society of Tropical Medicine and Hygiene 101, 1253–1264. 10.1016/j.trstmh.2005.03.018 17905372

[24] IBM Corp. Released (2021) *IBM SPSS Statistics for Windows, Version 28.0.* Armonk, NY: IBM Corp.

[25] Statistics Finland’s free-of-charge databases (n.d.) https://pxdata.stat.fi/PxWeb/pxweb/en/Kuntien_avainluvut/ (accessed 1 August 2023).

[26] Ruo SL, et al. (1991) Antigenic relatedness between arenaviruses defined at the epitope level by monoclonal antibodies. Journal of General Virology 72, 549–555. 1706408 10.1099/0022-1317-72-3-549

[27] Wilson MR and Peters CJ (2014) Diseases of the central nervous system caused by lymphocytic choriomeningitis virus and other arenaviruses. In Tselis AC and Booss J (eds), Handbook of Clinical Neurology, 3rd ed. Elsevier, vol. 123, pp. 671–681. https://www.sciencedirect.com/science/article/abs/pii/B978044453488000033X?via=ihub. 25015511 10.1016/B978-0-444-53488-0.00033-X

[28] Bond N, et al. (2013) A historical look at the first reported cases of Lassa fever: IgG antibodies 40 years after acute infection. American Journal of Tropical Medicine and Hygiene 88, 241–244. 23390223 10.4269/ajtmh.2012.12-0466 PMC3583312

[29] Alburkat H, et al. (2020) Lymphocytic Choriomeningitis virus infections and Seroprevalence, Southern Iraq. Emerging Infectious Diseases 26, 3002–3006. 33219805 10.3201/eid2612.201792 PMC7706927

[30] Finnish Biodiversity Info Facility (n.d.) https://laji.fi/en/observation/map?target=MX.48778 (accessed 17 January 2023).

