# Supplementary material for: Serological and molecular screening of arenaviruses in suspected tick-borne encephalitis cases in Finland
Source: Epidemiol Infect. 2024 Jan 22;152:e20. doi: 10.1017/S0950268824000128 (PMC10894894; doi:10.1017/S0950268824000128)

Serological and molecular screening of arenaviruses in suspected TBE cases in Finland

Alburkat H^1,2*^, Pulkkinen E^3,4^, Virtanen J^1,2^, Vapalahti O^1,2,3^, Sironen T^1,2^, Jääskeläinen AJ^3^

**Supplementary Material**

Supplementary Figure: Supplementary Figure S1: Distribution of age grouped by geographical region (A) and IgG status (B). Statistically significant differences (p<0.05) are indicated above the picture.


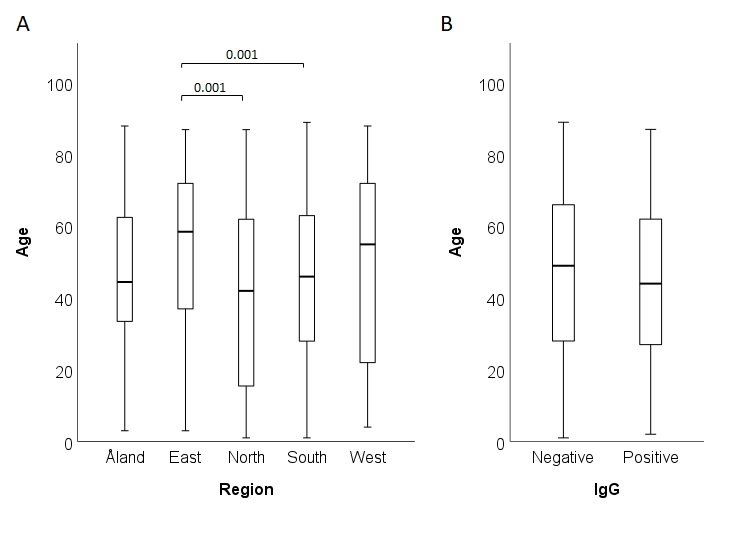

Supplement: Alburkat et al. supplementary material 1 — Alburkat et al. supplementary material [file S0950268824000128sup001.docx]
