# Supplementary material for: Serological and molecular screening of arenaviruses in suspected tick-borne encephalitis cases in Finland
Source: Epidemiol Infect. 2024 Jan 22;152:e20. doi: 10.1017/S0950268824000128 (PMC10894894; doi:10.1017/S0950268824000128)
Supplement: Alburkat et al. supplementary material 2 — Alburkat et al. supplementary material [file S0950268824000128sup002.docx]

Serological and molecular screening of arenaviruses in suspected tick-borne encephalitis cases in Finland

Alburkat H^1,2*^, Pulkkinen E^3,4^, Virtanen J^1,2^, Vapalahti O^1,2,3^, Sironen T^1,2^, Jääskeläinen AJ^3^

**Supplementary Material**

Supplementary table: Statistical tests for the age and seroprevalence distribution between the regions and individuals.

| **Is Age normally distributed** | | |
| --- | --- | --- |
| Shapiro-wilk test | Statistic | 0.961 |
|  | Sig.* | <0.001 |
|  | N | 867 |
| **Is age similar in different regions** | | |
| Independent-Samples Kruskal-Wallis test | N | 867 |
|  | Adj. Sig | 19.884 |
|  | Test statistic | <0.001 |
| Pairwise comparison (adj. Sig/std. test statistic) | North-South | 1.000/-1.183 |
|  | North-Åland | 1.000/-1.229 |
|  | North-West | 0.539/-1.928 |
|  | North-East | 0.001/4.000 |
|  | South-Åland | 1.000/-0.506 |
|  | South-West | 1.000/-1.361 |
|  | South-East | 0.001/3.803 |
|  | Åland-West | 1.000/0.736 |
|  | Åland-East | 0.342/1.089 |
|  | West-East | 1.000/1.089 |
| **Is there a difference in age between seropositive and negative individuals** | | |
| Independent-Samples Mann-Whitney U-test | N | 867 |
|  | Stand. test statistic | -0.731 |
|  | Exact Sig. | 0.465 |
| **Is the seroprevalence the same in different locations** | | |
| Fisher-Freeman-Halton exact test | Value | 7.851 |
|  | Exact Sig. | 0.080 |
| *All p-values are two-sided | | |
